# Modulation of the Dental Pulp Stem Cell Secretory Profile by Hypoxia Induction Using Cobalt Chloride

**DOI:** 10.3390/jpm11040247

**Published:** 2021-03-30

**Authors:** Shilpa Bhandi, Ahmed Al Kahtani, Mohammed Mashyakhy, Loai Alsofi, Prabhadevi C. Maganur, Satish Vishwanathaiah, Luca Testarelli, Andrea Del Giudice, Deepak Mehta, Nishant Vyas, Vikrant R. Patil, A. Thirumal Raj, Shankargouda Patil

**Affiliations:** 1 Department of Restorative Dental Sciences, College of Dentistry, Jazan University, Jazan 45142, Saudi Arabia; shilpa.bhandi@gmail.com (S.B.); dr.mashyakhy@gmail.com (M.M.); 2 Department of Restorative Dental Sciences, College of Dentistry, King Saud University, Riyadh 11451, Saudi Arabia; ahkahtani@ksu.edu.sa; 3 Department of Endodontics, Faculty of Dentistry, King Abdulaziz University, Jeddah 21589, Saudi Arabia; lalsofi@kau.edu.sa; 4 Department of Preventive Dental Sciences, Division of Pedodontics, College of Dentistry, Jazan University, Jazan 45142, Saudi Arabia; prabhadevi.maganur@gmail.com (P.C.M.); drvsatish77@gmail.com (S.V.); 5 Department of Oral and Maxillofacial Sciences, “Sapienza” University of Rome, 00185 Rome, Italy; luca.testarelli@uniroma1.it (L.T.); andrea.delgiudice@uniroma1.it (A.D.G.); 6 Department of Preventive and Restorative Dentistry, College of Dental Medicine, University of Sharjah, Sharjah 27272, United Arab Emirates; dmehta@sharjah.ac.ae; 7 Logical Life Science Private Limited, Pune 411041, India; logicalbiology84@gmail.com (N.V.); patilvikrant.r@gmail.com (V.R.P.); 8 Department of Oral Pathology and Microbiology, Sri Venkateswara Dental College and Hospital, Chennai 600130, India; thirumalraj666@gmail.com; 9 Department of Maxillofacial Surgery and Diagnostic Sciences, Division of Oral Pathology, College of Dentistry, Jazan University, Jazan 45142, Saudi Arabia

**Keywords:** cobalt chloride, dental pulp stem cells, hypoxia inducible factor, secretory profile, stemness

## Abstract

The action of stem cells is mediated by their paracrine secretions which comprise the secretory profile. Various approaches can be used to modify the secretory profile of stem cells. Creating a hypoxic environment is one method. The present study aims to demonstrate the influence of CoCl_2_ in generating hypoxic conditions in a dental pulp stem cell (DPSCs) culture, and the effect of this environment on their secretory profile. DPSCs that were isolated from human permanent teeth were characterized and treated with different concentrations of CoCl_2_ to assess their viability by an 3-(4,5-dimethylthiazol-2-yl)-2,5-diphenyltetrazolium bromide (MTT) assay and proliferation by a cell counting kit (CCK)-8 assay. The gene expression level of hypoxia-inducible factor 1-alpha (HIF-1α) was analyzed by quantitative real time polymerase chain reaction (qRT-PCR) to demonstrate a hypoxic environment. Comparative evaluation of the growth factors and cytokines were done by cytometric bead array. Gene expression levels of transcription factors OCT4 and SOX2 were analyzed by qRT-PCR to understand the effect of CoCl_2_ on stemness in DPSCs. DPSCs were positive for MSC-specific markers. Doses of CoCl_2,_ up to 20 µM, did not negatively affect cell viability; in low doses (5 µM), it promoted cell survival. Treatment with 10 µM of CoCl_2_ significantly augmented the genetic expression of HIF-1α. Cells treated with 10 µM of CoCl_2_ showed changes in the levels of growth factors and cytokines produced. It was very evident that CoCl_2_ also increased the expression of OCT4 and SOX2, which is the modulation of stemness of DPSCs. A CoCl_2_ treatment-induced hypoxic environment modulates the secretory profile of DPSCs.

## 1. Introduction

The dental pulp forms the core of every vital tooth. It has a nutritive, formative, and reparative function. To fulfill these functions, it has a complex architecture and contains numerous cells and tissues such as lymphatics, neural fibres, connective tissue, blood vessels, and stem cells. Stem cells are pluripotent cells capable of giving rise to specialized cells in the body. The ones present in the dental pulp, also known as dental pulp stem cells (DPSCs), are a kind of mesenchymal stem cell (MSCs). They were first described, as well as characterized, by Gronthos et al. in 2000 and were found to be comparable to bone marrow stem cells [1]. DPSCs express stem cell markers such as KLF4, OCT4, SOX2, SSEA-3, SSEA-4, and Nanog [2,3]. They respond by separating into specialized cells if the dental pulp suffers an insult. They give rise to dentin-forming cells, i.e., odontoblasts, when they are damaged due to an external stimulus to help maintain the integrity of the pulp. DPSCs also have adipogenic, osteogenic, chondrogenic, myogenic, and neurogenic potential [2,4,5]. DPSCs offer the advantage of being isolated by non-invasive methods from their source such as being extracted from wisdom teeth. They can be cryopreserved and still retain their multipotent characteristics [5,6,7]. They have applications in regenerative endodontic therapy, bone regeneration, formation of neural tissues, healing, angiogenesis, cartilage synthesis, and immunomodulation [8]. DPSCs have also been used as carriers for the targeted delivery of the anti-cancer drug, paclitaxel, making them a potential therapeutic option that can reduce morbidity from chemotherapy [9].

The therapeutic action of stem cells is not solely a result of the replacement of dead or injured cells. After intravenous, intra-arterial, or intra-peritoneal infusion, most mesenchymal stem cells are entrapped in the lungs after 48 h [10]. Therapeutic effects of stem cells are seen regardless of cell migration to affected areas. This is attributed to the paracrine actions of MSCs. All living cells produce proteins within the extracellular space, which include enzymes, chemokines, cytokines, adhesion molecules, and growth factors. The collective term for these extracellular secretions is secretory profile [11,12]. MSC secretory profiles have heterogeneity in different populations and possess therapeutic potential for many diseases [11]. A DPSC secretory profile has a positive effect on the viability of cells, helps protect against cytotoxicity, and enhances the potential for repair and mineralization [13]. The secretory profile from stem cells obtained from deciduous teeth has been used for the treatment of osteoarthritis in the temporomandibular joint of a mouse model [14].

Various approaches can be used to modify the secretory profile of MSCs. These include molecular priming, hypoxic preconditioning, tissue engineering, and growth media composition [11]. Of all the mentioned approaches, hypoxia is of interest as the local oxygen concentration in the tissues where an MSCs resides is below normal [15,16]. The dental pulp also has a hypoxic environment in animal models [17,18]. In vitro studies on mesenchymal stem cells are performed at normal oxygen concentrations. In contrast, the in vivo conditions where the cells and their products find application have hypoxic conditions. It has therefore been suggested that hypoxic preconditioning should be made a standardized procedure for MSCs before clinical use [19].

Hypoxic conditions can be created either physically, i.e., using a hypoxic chamber with control of the oxygen levels, or chemically, by using chemicals in the culture media. The methods requiring physical control of oxygen levels require constant monitoring and specialized equipment making the use of chemicals such as cobalt chloride (CoCl_2_) or dimethyloxalylglycine (DMOG) a more favourable choice [20]. CoCl_2_ has been used several times to induce and mimic hypoxic conditions, in vitro [21].

The hypoxia inducible factor (HIF) is a transcription factor with an α subunit (-1α, -2α and -3α), which is oxygen controlled, and a β subunit [22]. The subunit responsible for optimizing respiration under hypoxic conditions is HIF-1α. It is constantly synthesized and degraded in normoxic conditions but stabilizes in a hypoxic environment. CoCl_2_ stabilizes HIF-1α under normoxic conditions, inducing chemical hypoxia which lasts several hours [21]. This has utility in studying stem cell markers and osteogenic differentiation in human DPSCs (hDPSCs) and periodontal cells [23,24,25]. However, the effect of these hypoxic conditions on the secretory profile has not been evaluated in detail.

The objective of this study was to evaluate the influence of CoCl_2_ induced hypoxia on the secretory profile of human dental pulp stem cells as well as on the stemness of the same. We specifically aimed to study the effect of this condition on the cytokines and growth factors in the secretions of hDPSCs and the effects and expression levels of OCT4 and SOX2, the stemness transcription factors. The potential clinical relevance for the present study was to assess if inducing a hypoxic state could maximize the regenerative potential of hDPSCs. Augmented stemness through hypoxia could aid in prolonging/potentiating the hDPSCs regenerative potential. Based on the results of the present study, future studies could use hypoxia as an optimizing condition for eliciting augmented proliferative and differentiative properties.

## 2. Materials and Methods

### 2.1. Sample Collection

Samples were obtained from extracted human premolar teeth from healthy subjects aged 18–25 after obtaining their consent. The extractions were carried out for orthodontic treatment. 5 premolar teeth were collected from patients with good oral hygiene. The pulp was isolated under sterile conditions and transferred to the laboratory for further processing.

### 2.2. Isolation and Culture of DPSCs

DPSCs were isolated using the long-term explant culture technique which has been described in detail in another paper [26]. Small fragments of the pulp tissue, measuring ~1 mm, were placed in culture flasks and covered completely in fetal bovine serum (FBS) and incubated. The explant was sustained in a Dulbecco’s Modified Eagle’s Medium (DMEM) of 20% fetal bovine serum and supplementary antibiotic-antimycotic solution. It was maintained in a humid environment of 5% CO_2_ at a temperature of 37 °C. The culture medium was renewed every 2–3 days. Cellular outgrowth was monitored regularly with an inverted phase-contrast microscope. Cells that were outgrown with a 70–80% confluence were removed from the flasks by trypsinization and transferred to another flask. The primary explant culture was maintained. DPSCs obtained at passage 4 were used characterized and studied.

### 2.3. Characterization Using Flow Cytometry

Confluent DPSCs harvested from the flask by trypsinization were washed with phosphate-buffered saline. After incubating in the dark at room temperature for thirty minutes with human leukocyte antigen—DR isotype (HLA-DR), CD73, CD90, and CD105 antibodies, phosphate-buffered saline was used to wash the cells. They were then analyzed on a flow cytometer. No less than ten thousand events were acquired per sample. The extent of positive staining was taken as a percentage to compare to the controls.

### 2.4. 3-(4,5-Dimethylthiazol-2-yl)-2,5-Diphenyltetrazolium Bromide (MTT) Assay

This assay checked the viability of DPSCs treated with the different concentrations of CoCl_2_. A control group without treatment, and cells treated with concentrations of 5, 10, 20, and 40 µM were used. After being seeded into 96 well plates, the cells were incubated with culture media and CoCl_2_ for 48 h. Following this, the MTT solution of a 0.5 mg/mL concentration was placed in individual wells, and these were incubated for 4 h at 37 °C. Subsequently, the media was replaced with 100 µL of dimethyl sulfoxide in every well. A spectrophotometry plate reader was used to evaluate the absorbance seen at 570 nm.

### 2.5. Cell Proliferation Assay CCK-8 (Cell Counting Kit 8 Assay)

This assay was done, at 1 to 10 days, in treatments with CoCl_2_ (10 µM) and in the control group with no treatment. CCK-8 is a colorimetric reaction-based assay that yields an orange formazan dye to an extent proportional with the cell number. The proliferation rate of DPSCs was calculated by evaluating the absorbance at 450 nm on a spectrophotometer.

### 2.6. Quantitative Real-Time PCR for Analysis of Gene Expression

For this test, a complementary DNA (cDNA) synthesis kit was used to reverse transcribe 1 µg of total RNA, as per manufacturer guidelines. Quantitative analysis of the gene of interest (HIF-1α) was done using the SYBR Green PCR master mix, using a qRT-PCR machine, in treated (10 µM CoCl_2_) and untreated groups. Normalization of the expressions of target genes to ß-actin was done by using the ΔΔCt technique. The quantification of data was done by using the 2^–ΔΔCt^ technique, and indicated as a normalized relative expression of the gene compared to the average of the β-actin gene CT value. The utilized primer sequences are mentioned in Table 1.

### 2.7. Cytometric Bead Array for the Detection of Cytokines and Growth Factors

For evaluating the levels of the cytokines and growth factors in the conditioned media for DPSCs, with and without treatment using CoCl_2,_ a cytometric bead array was done. A LEGENDplex™ Human Growth Factor Panel (13-plex) (Biolegend; 740180 San Diego, CA, USA) (VEGF, Ang-2, EGF, EPO, FGF-basic, PDGF-AA, PDGF-BB, G-CSF, GM-CSF, HGF, M-CSF, SCF, TGF-α) was used for the detection of the growth factors. A LEGENDplex™ Human Essential Immune Response Panel (13-plex) (Biolegend; 740929) (IL 4, IL 2, IL 1β, IL 17A, IL 6, IL 10, IL 12p70, TNF α, CXCL8, CXCL10, CCL2, IFN γ, TGF β1) was used for the detection of the cytokines. The experimental protocol was performed as per the manufacturer’s guidelines. Briefly, 25 μL of the conditioned media was incubated with the microbeads, and detection antibodies were subsequently introduced. The samples were then evaluated using a flow cytometer and analysis was performed by using LEGENDplex™ Data Analysis Software.

### 2.8. Statistical Analysis

All the samples were experimented in triplicate. All the experimental protocols were performed three times. Means and standard deviations of all the experimental values for each sample were used to represent the data for five individual samples. The statistical significances between the experimental groups were determined by a paired *t*-test (two-tailed) and *p*-values were calculated using the GraphPad Prism 8 software (GraphPad Software, La Jolla, CA, USA). A *p*-value of less than 0.05 was measured as significant (Significance Levels: ns not significant, * *p* < 0.05, and ** *p* < 0.01).

## 3. Results

### 3.1. DPSC Characterization

**Figure 1:**
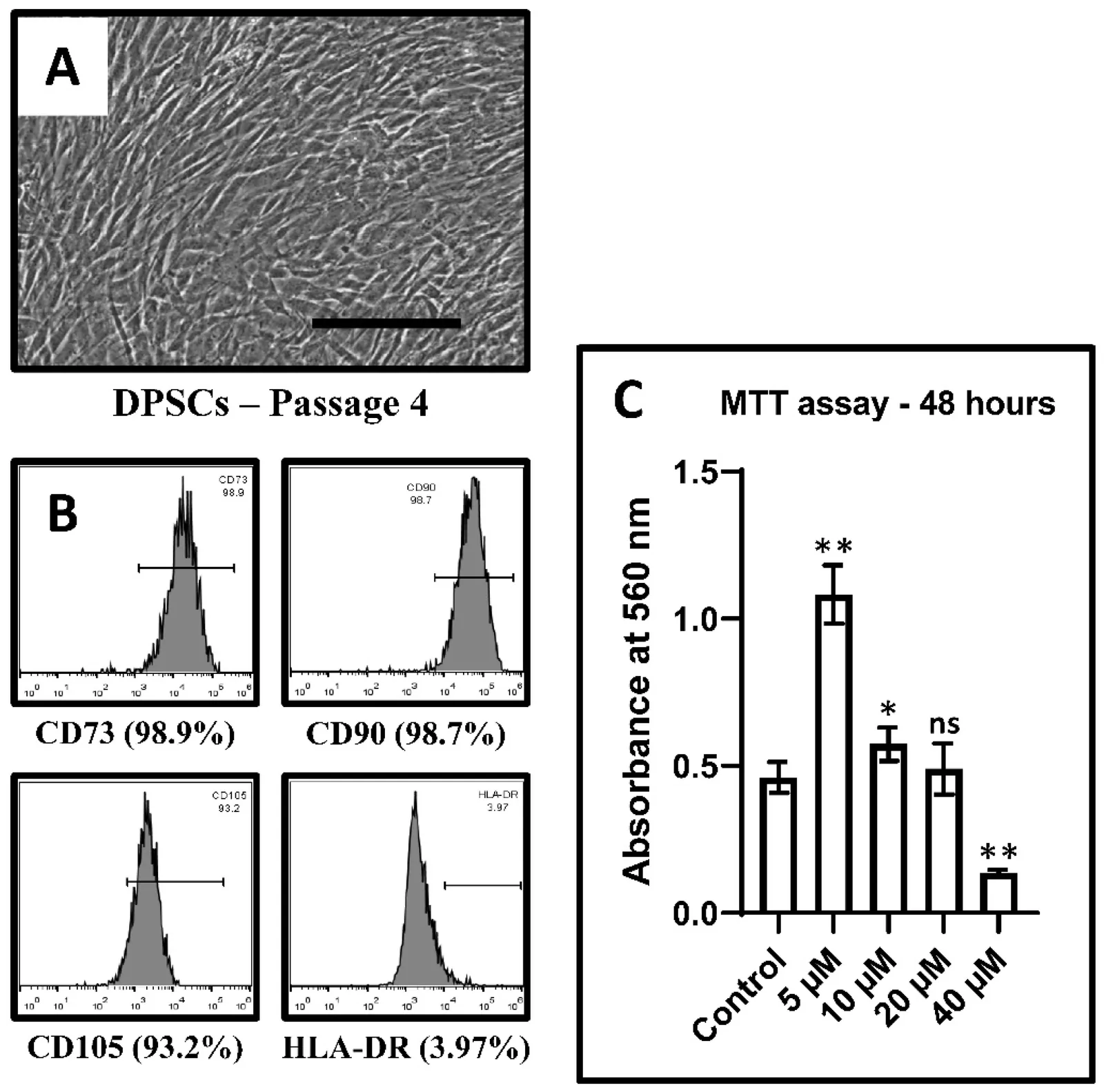
(**A**): elongated spindle-shaped morphology of the isolated cells; (**B**): MHC II class representation; (**C**): cell viability at high concentration of CoCl_2_. ns not significant, * *p* < 0.05, and ** *p* < 0.01.

Morphological evaluation, done using the inverted phase contrast microscope, showed an elongated spindle-shaped morphology of the isolated cells (Figure 1A). The cells displayed mesenchymal stem cell-specific markers CD73, CD90, and CD105. The MHC Class II cell surface receptor was absent in the cultured cells (Figure 1B). Thus, the DPSCs had an MSC-like morphology and stem cell surface markers.

### 3.2. Effect of CoCl_2_ on Proliferation and Viability

After 48 h, no difference in cellular metabolism was observed by the MTT assay. However, a low dose of 5 µM showed a significant increase in cell metabolism, which was the highest of all the concentrations used. The cell viability was lowest at a high concentration of CoCl_2_ (40 µM) after 48 h (Figure 1C) (Table 2). Cell viability was significantly unaffected by the CoCl_2_ treatment, for concentrations of 10 and 20 µM.

**Figure 2:**
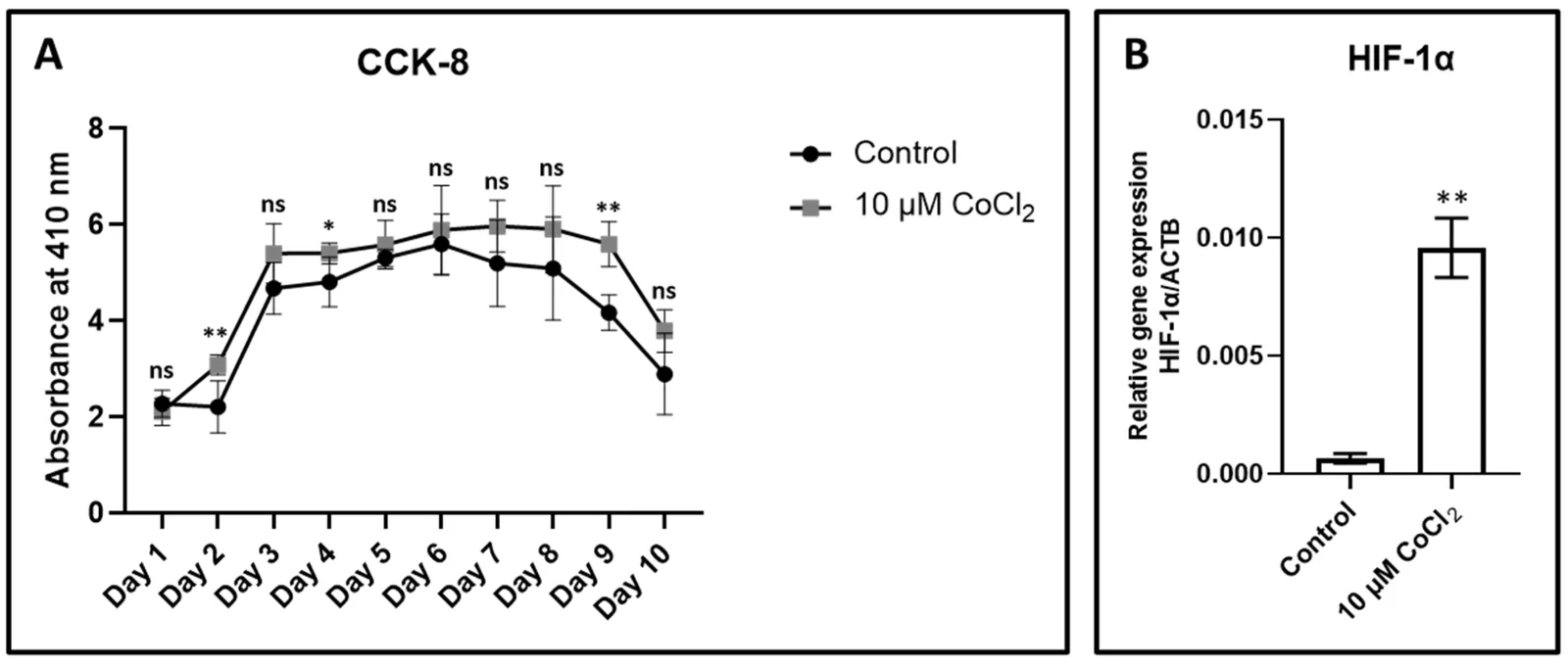
(**A**)**:** Chronological evaluation of absorbability; (**B**)**:** PCR results. ns not significant, * *p* < 0.05, and ** *p* < 0.01.

Cell proliferation measured by the CCK–8 Assay showed a higher absorbance of cells treated with CoCL_2_ (10 µM) at all times. However, the results were significant only on day 9. (Figure 2A) (Table 3). Thus, CoCL_2_ had a positive effect on cell viability at low concentrations and proliferation was also higher upon treatment.

### 3.3. Expression of Hypoxia-Inducing Gene HIF-1α

A significant upregulation of HIF -1α, after treatment with 10 µM CoCl_2_ compared to an untreated group, was observed in the quantitative real time PCR results (Figure 2B) (Table 4).

### 3.4. Modulation of the Secretory Profile of DPSCs in Terms of Growth Factors and Cytokines

**Figure 3:**
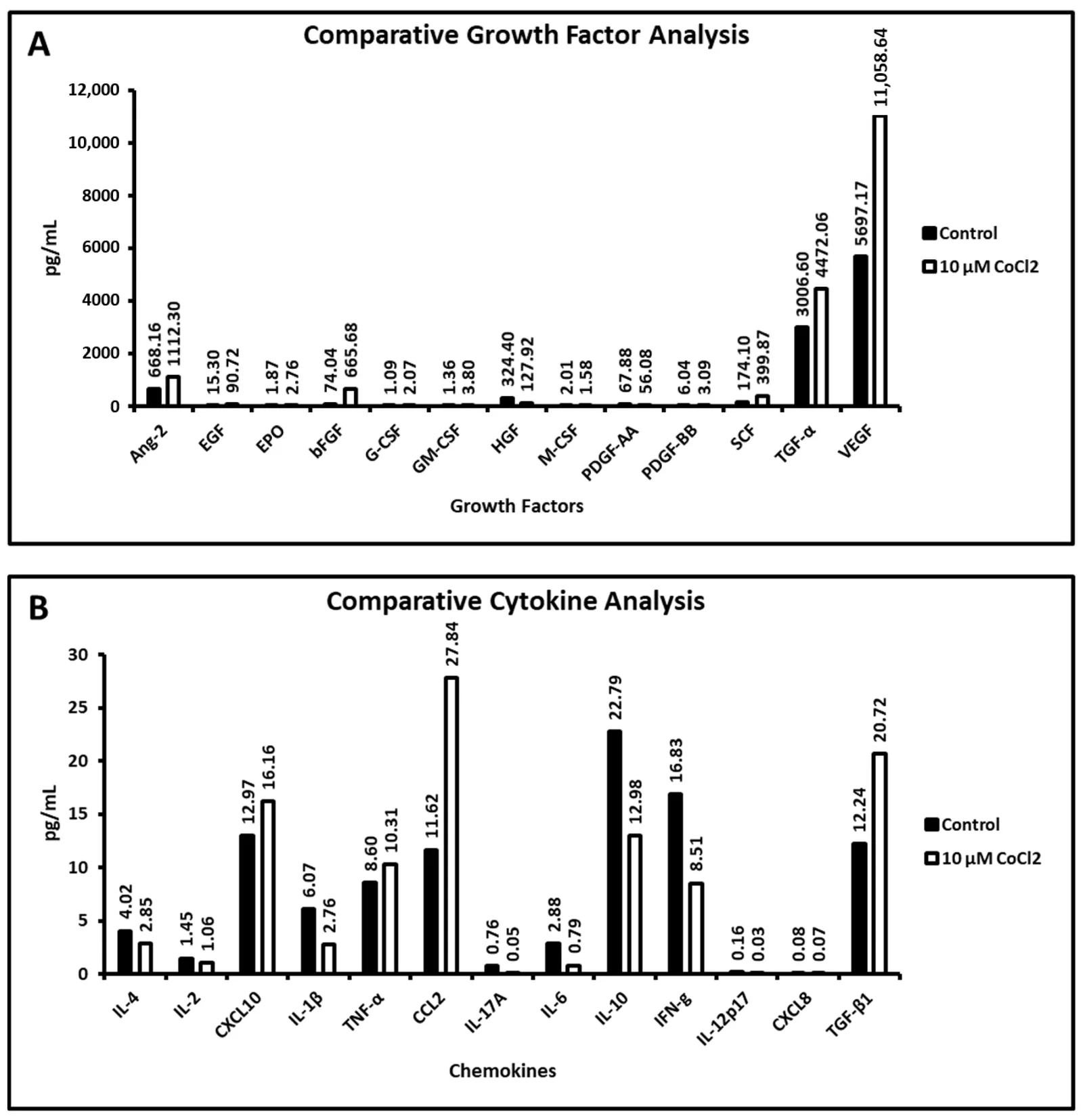
(**A**)**:** growth factor distribution; (**B**)**:** Inflammatory cytokines distribution.

A comparative analysis of the cytokines and growth factors in the conditioned-media from DPSCs and CoCl_2_-treated DPSCs showed that the growth factors, especially those associated with angiogenesis were higher in the secretory profile of CoCl_2_-treated DPSCs. These molecules include Ang-2, EGF, bFGF, SCF, and VEGF (Figure 3A) (Table 5). Another observation was higher levels of the anti-inflammatory cytokines CCL2, CXCL10, and TGF–β1, and reduced secretion of interferon gamma and interleukins 4, 6, and 10 by DPSCs treated with CoCl_2_ (Figure 3B) (Table 6).

### 3.5. Effect of CoCl2 Induced Hypoxia on Gene Expression of OCT4 and SOX2

**Figure 4:**
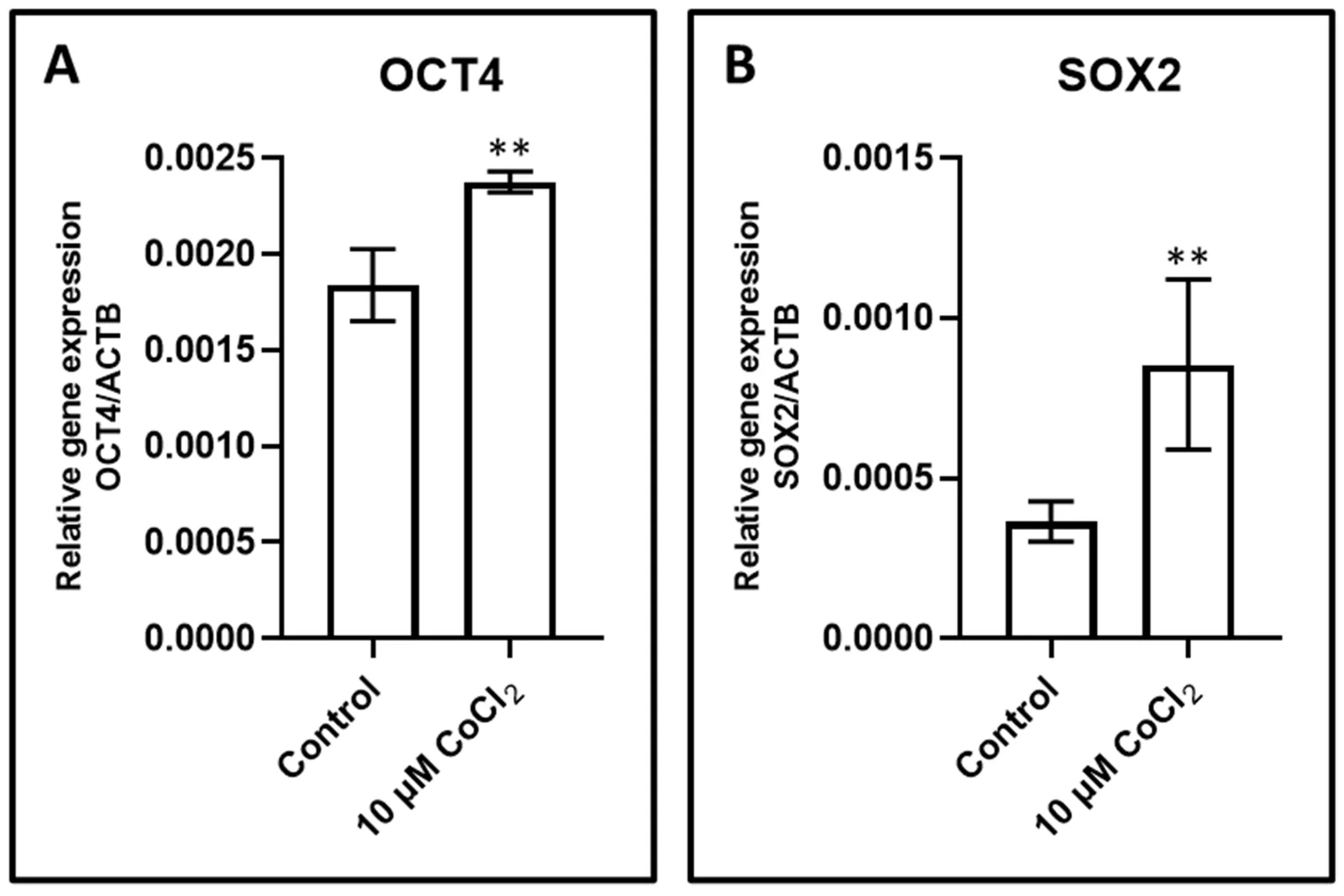
(**A**)**:** OCT4 levels; (**B**): SOX2 levels. ns not significant, ** *p* < 0.01.

The CoCl_2_ treated and untreated DPSCs were studied for the gene expression of stemness transcription factors OCT4 and SOX2. 10 μM of CoCl_2_ treated DPSCs showed elevated levels of OCT4 and SOX2 expression levels, with respect to the mRNA when compared with untreated cells (Figure 4A,B) (Table 7).

## 4. Discussion

In this study, we aimed to demonstrate that hypoxic conditions can modify the secretory profile of DPSCs. As observed in previous studies, DPSCs have characteristics similar to mesenchymal stem cells which were also seen in this study [1,4]. These similarities betweeen the DPSCs and MSCs are maintained in vivo as well [27]. The lack of MHC receptors indicates that these cells might have use as allogenic sources in vivo without inducing an immune response. Hypoxic conditions are known to affect mesenchymal stem cells. They have demonstrated positive effects on cell proliferation and surface markers for MSCs [16,28,29]. Similar results have been observed in DPSCs as well [23,24]. In DPSCs, hypoxic conditions were able to enhance the angiogenic activity and decreased the osteogenic differentiation of these cells [23,30,31].

CoCl_2_ is a mimetic hypoxia agent, like many others. It has been used to induce chemical hypoxia for studying stem cells. It has demonstrated responses similar to those produced by cells subjected to hypoxia [21,32]. The mechanism of action that CoCl_2_ uses in producing a hypoxic environment is attributed to the substitution of iron by cobalt ions in prolyl hydroxylases (PHDs). These enzymes are responsible for the degradation of the hypoxia induced factor in normal oxygen concentrations. CoCl_2_ thus stabilizes the HIF expression and creates a response similar to a hypoxic condition. Various concentrations of CoCl_2_ have been used to study stem cells. High concentrations of 100 µM have been shown to induce cell death in dental pulp stem cells, while the same concentration does not affect periodontal cells [23,25]. Even in MSCs, the effect of CoCl_2_ is debated with conflicting results in the studies [20,33]. These conflicting results may be obtained due to the varying influence of CoCl_2_ depending on the cell type and the concentration of the solution used. Further research in this regard can help identify the ideal conditions for studying different cell types.

HIF 1 regulates the haemostatic response to hypoxia. An increase in the hypoxia inducible factor is the foremost response in cells under hypoxic stress [34]. It acts by reprogramming the glycolytic metabolism and inducing genes encoding phosphofructokinase, pyruvate kinase, and hexokinase, and glucose transporters 1 and 3 [35]. Another effect of HIF is the upregulation of gene encoding proteins involved in angiogenesis and erythropoiesis [36]. Although CoCl_2_ is known to induce hypoxia-like conditions via stabilization of HIF, there are other factors such as the physical presence of oxygen ions, which make it difficult to attribute the extent of the observed effects to hypoxia.

Regarding stem cells, hypoxia has been proven to be essential for the upkeep of stem cell properties, that is their multipotency, self-renewal, and survival [37]. However, the influence of hypoxia on the secretory profile of stem cells of the dental pulp, needs further research. This study shows that hypoxic conditions do affect the secretory profile of dental pulp stem cells. There was an increase in angiogenic cytokines which can be expected with an upregulation of HIF 1α. Growth factors such as EPO, Ang -2, bFGF, SCF, TGF α, and VEGF were increased (Figure 3A). This indicates an enhanced anabolic potential in the treated cells compared to controls. The hypoxia primed cells will probably be more effective and efficient than untreated controls in affecting therapeutic changes. The cytokine secretion was also modulated by hypoxia. The levels of certain chemokines CXCL10 and CCL2 were higher whereas interleukins were reduced. This indicates that the hypoxic condition promotes cell signaling and communication, but at the same time reduces pro-inflammatory activity. The overall effect of the chemical hypoxia appears to be an increase in synthetic activity and reduced inflammation in the DPSC secretory profile.

The effect of hypoxia on the secretory profile of stem cells has been studied before. In a 3-D culture system, DPSCs under hypoxia showed an elevated secretion of IL-6 [38]. In another study on a mesenchymal stem cell sheet, secretions of VEGF, bFGF and collagen I and III were enhanced under hypoxic conditions [39]. Along with an enhanced proliferation, the chemokine receptor (CXCR4) has also been demonstrated to be elevated on DPSCs under hypoxia [40]. The increased secretion of these pro-angiogenic and anti-inflammatory factors is what could provide a potential therapeutic application to DPSCs. They have been used in the form of nanofibrous microspheres to aid in the regeneration of the pulp [41].

OCT4 and SOX2 are the transcription factors which define the stemness of the stem cells. Both of these transcription factors control various epigenetic processes, molecular regulatory pathways, and even the proliferation in stem cells. In the current study the hypoxia induced by CoCl_2_ increased the gene expression of both OCT4 and SOX2 (Figure 4A,B), it is a very good indication for potential new directions of research where the stemness and the quality of DPSCs can be increased by chemically induced hypoxia.

To be used as therapeutic agents, stem cells must be easily harvested and provide desired benefits, with minimal adverse effects. Dental stem cells obtained from various sources are helpful in this regard. The secretory profile of stem cells plays a major role in their action. Various approaches in modifying the secretory profile need to be researched to provide the appropriate molecular therapy for a disease. Inducing hypoxia, as in the present study, has been shown to augment DPMSC regenerative properties, including osteogenic and angiogenic potential, as elicited by Maslowska et al. [42] and Dissanayaka et al. [43]. Similar studies on various molecules that bring about the modification will help to better understand the mechanism and develop protocols for future medicine. However, this study should be further expanded to examine the effect of hypoxia induction on the differentiation of DPSCs in multiple lineages, clonogenic aptitude, and cellular senescence. Moreover, the paracrine effect of DPSCs primed with a hypoxic condition need to be assessed for their immunomodulatory and regenerative potential in terms of allogeneic transplantation and wound healing ability. Furthermore, synergistic effects of hypoxia, along with some pharmaceutical conditionings, need to be studied to fill the lacunae in empowerment of DPSCs for therapeutic use. Finally, a high throughput and sophisticated experimentation should be utilized to excavate the maximum clinical efficacy of DPSCs.

## 5. Conclusions

This study demonstrates that CoCl_2_ can induce a hypoxia-like environment for DPSCs by stabilization of the HIF 1α. The hypoxic environment modified the secretory profile of the DPSCs by increasing the secretion of synthetic factors and reduced the secretion of inflammatory mediators. Moreover, the stemness-related transcription factors were also found to be upregulated by CoCl_2_. This demonstrates an increased potential for therapeutic purposes in hypoxia primed cells over untreated cells.

## Figures and Tables

**Table 1:** Primer Sequences used for PCR.

Gene	Forward Primer	Reverse Primer
HIF-1α	5′-CTT CTG AGC TCT GAT GAG GC-3′	5′-GAA AGC ACC ATC AGG AAG CC-3′
OCT4	5′-TTT TGG TAC CCC AGG CTA TG-3′	5′-GCA GGC ACC TCA GTT TGA AT-3′
SOX2	5′-GAG CTT TGC AGG AAG TTT GC-3′	5′-GCA AGA AGC CTC TCC TTG AA-3′
ACTB	5′-AGA GCT ACG AGC TGC CTG AC-3′	5′-AGC ACT GTG TTG GCG TAC AG-3′

**Table 2:** Effect of CoCl_2_ on viability.

CoCl_2_ Treatment	Control	5 µM	10 µM	20 µM	40 µM
Absorbance at 560 nm	0.46 ± 0.052	1.082 ± 0.098	0.57 ± 0.056	0.49 ± 0.086	0.13 ± 0.010
*p* value (vs. Control)		<0.0001	0.0116	0.5352	<0.0001

**Table 3:** Effect of CoCl_2_ on proliferation.

CCK-8 Assay	Control	10 µM CoCl_2_	*p* Value (vs. Control)
Day 1	2.27 ± 0.27	2.10 ± 0.28	0.3709
Day 2	2.21 ± 0.54	3.08 ± 0.21	0.0096
Day 3	4.67 ± 0.53	5.4 ± 0.62	0.0853
Day 4	4.81 ± 0.52	5.41 ± 0.21	0.0449
Day 5	5.31 ± 0.18	5.58 ± 0.50	0.2820
Day 6	5.59 ± 0.63	5.88 ± 0.92	0.5721
Day 7	5.19 ± 0.90	5.97 ± 0.53	0.1391
Day 8	5.08 ± 1.07	5.91 ± 0.90	0.2270
Day 9	4.16 ± 0.36	5.59 ± 0.46	0.0007
Day 10	2.89 ± 0.84	3.78 ± 0.44	0.0685

**Table 4:** Effect of CoCl_2_ on HIF -1α expression.

Relative Gene Expression/ACTB	Control	10 µM CoCl_2_	*p* Value (vs. Control)
HIF-1α	0.00065 ± 0.000019	0.0095 ± 0.0012	<0.0001

**Table 5:** Effect of CoCl_2_ on growth factor secretion by DPSCs.

Growth Factors	Control	10 µM CoCl_2_
Ang-2	668.16	1112.30
EGF	15.29	90.72
EPO	1.87	2.76
bFGF	74.04	665.68
G-CSF	1.09	2.07
GM-CSF	1.36	3.80
HGF	324.40	127.92
M-CSF	2.01	1.58
PDGF-AA	67.88	56.08
PDGF-BB	6.04	3.09
SCF	174.10	399.87
TGF-α	3006.60	4472.06
VEGF	5697.17	11,058.64

**Table 6:** Effect of CoCl_2_ on cytokine secretion by DPSCs.

Cytokines	Control	10 µM CoCl_2_
IL-4	4.02	2.85
IL-2	1.45	1.06
CXCL10	12.97	16.16
IL-1β	6.07	2.76
TNF-α	8.59	10.30
CCL2	11.62	27.84
IL-17A	0.76	0.05
IL-6	2.85	0.79
IL-10	22.79	12.97
IFN-g	16.83	8.51
IL-12p17	0.16	0.03
CXCL8	0.08	0.07
TGF-β1	12.23	20.72

**Table 7:** Effect of CoCl_2_ on stemness related genes OCT4 and SOX2 expression in DPSCs.

Relative Gene Expression/ACTB	Control	10 µM CoCl_2_	*p* Value (vs. Control)
OCT4	0.0018 ± 0.00019	0.0023 ± 0.000054	0.0003
SOX2	0.00036 ± 0.000062	0.00084 ± 0.00026	0.0039

## Data Availability

The data presented in this study are available on request from the corresponding author.
