# Correction: Bhandi et al. Modulation of the Dental Pulp Stem Cell Secretory Profile by Hypoxia Induction Using Cobalt Chloride. *J. Pers. Med.* 2021, *11*, 247

**DOI:** 10.3390/jpm16070381

**Published:** 2026-07-17

**Authors:** Shilpa Bhandi, Ahmed Al Kahtani, Mohammed Mashyakhy, Loai Alsofi, Prabhadevi C. Maganur, Satish Vishwanathaiah, Luca Testarelli, Andrea Del Giudice, Deepak Mehta, Nishant Vyas, Vikrant R. Patil, A. Thirumal Raj, Shankargouda Patil

**Affiliations:** 1 Department of Restorative Dental Sciences, College of Dentistry, Jazan University, Jazan 45142, Saudi Arabia; 2 Department of Restorative Dental Sciences, College of Dentistry, King Saud University, Riyadh 11451, Saudi Arabia; 3 Department of Endodontics, Faculty of Dentistry, King Abdulaziz University, Jeddah 21589, Saudi Arabia; 4 Department of Preventive Dental Sciences, Division of Pedodontics, College of Dentistry, Jazan University, Jazan 45142, Saudi Arabia; 5 Department of Oral and Maxillofacial Sciences, “Sapienza” University of Rome, 00185 Rome, Italy; 6 Department of Preventive and Restorative Dentistry, College of Dental Medicine, University of Sharjah, Sharjah 27272, United Arab Emirates; 7 Logical Life Science Private Limited, Pune 411041, India; 8 Department of Oral Pathology and Microbiology, Sri Venkateswara Dental College and Hospital, Chennai 600130, India; 9 Department of Maxillofacial Surgery and Diagnostic Sciences, Division of Oral Pathology, College of Dentistry, Jazan University, Jazan 45142, Saudi Arabia

The Author Contribution section in the original publication [1] was incomplete, and the Conflicts of Interest statement was incorrect. The corrected Author Contribution and Conflicts of Interest statements appear here. Minor typographical errors in Affiliation 2 and Reference 18 have also been corrected.

**Author Contributions**

Conceptualization, S.B., A.A.K.; methodology, M.M., L.A.; software, P.C.M., S.V.; validation, L.T., A.D.G.; formal analysis, D.M., V.R.P., N.V.; investigation, A.T.R., S.P.; resources, S.B., A.A.K.; data curation, M.M., L.A.; writing—original draft preparation, S.B., A.A.K., M.M., L.A., P.C.M., S.V.; writing—review and editing, L.T., A.D.G., D.M., V.R.P., N.V., A.T.R., S.P.; visualization, P.C.M., S.V.; supervision, L.T., A.T.R., S.P.; project administration, S.B., A.A.K. All authors have read and agreed to the published version of the manuscript.

**Conflicts of Interest**

Authors Nishant Vyas and Vikrant R. Patil were employed by the company Logical Life Science Private Limited. The remaining authors declare that the research was conducted in the absence of any commercial or financial relationships that could be construed as a potential conflict of interest.

The authors state that the scientific conclusions are unaffected. This correction was approved by the Academic Editor. The original publication has also been updated.
